# Case Report: Late-onset liver cirrhosis in an elderly patient with FBN1-related Geleophysic Dysplasia

**DOI:** 10.3389/fendo.2026.1822591

**Published:** 2026-09-04

**Authors:** Yuqi Liu, Ying Wang, Xiaochun Teng, Xiaoli Wang

**Affiliations:** Department of Endocrinology and Metabolism, Institute of Endocrinology, NHC Key Laboratory of Diagnosis and Treatment of Thyroid Diseases, The First Hospital of China Medical University, Shenyang, China

**Keywords:** case report, FBN1 gene, Geleophysic dysplasia, gene muatation, liver cirrhosis

## Abstract

**Background:**

FBN1-related geleophysic dysplasia (GD2) is an ultrarare autosomal dominant disorder caused by dominant-negative missense variants in the TGFβ-binding protein-like domain 5 (TB5) of *FBN1*. It is characterized by severe short stature, brachydactyly, progressive joint stiffness, and cardiac valvular disease. Although hepatomegaly is known to occur in GD, progression to cirrhosis with portal hypertension in the elderly has never been documented.

**Case presentation:**

A 76-year-old Chinese man presented with a 1-year history of abdominal distension and was found to have liver cirrhosis during routine examination. He had no history of viral hepatitis, alcohol abuse, or other conventional liver disease risk factors. Physical examination revealed short stature (128 cm), brachydactyly, and mild scleral icterus. His 39-year-old daughter shared a similar skeletal phenotype and had a history of cardiac valve replacement. Laboratory tests showed moderate hyperbilirubinemia and mild thrombocytopenia with normal liver enzymes. Serological screening markers for viral hepatitis and autoimmune liver disease were all unremarkable. Liver elastography (15.5 kPa) and contrast-enhanced MRI confirmed cirrhosis with portal hypertension. Whole-genome sequencing identified a heterozygous pathogenic *FBN1* variant (c.5284G>A, p.Gly1762Ser) in both the proband and his daughter, confirming a diagnosis of GD. The patient was managed supportively and remained stable over 6 months of follow-up.

**Conclusion:**

To our knowledge, this is the first reported case of late-onset liver cirrhosis in an elderly GD patient caused by an *FBN1* variant. This case raises the possibility that hepatic manifestations related to GD could be linked to late-onset cirrhosis, though a causal or progressive relationship cannot be confirmed due to absent serial hepatic assessments prior to cirrhosis diagnosis. For elderly patients with unexplained late-onset liver cirrhosis combined with short stature or multisystem malformations, *FBN1* genetic testing may be considered. In addition, long-term hepatic monitoring may be considered for middle-aged and elderly patients or those with TB5 domain variants, although universal surveillance for all affected individuals is not supported by the current evidence.

## 1 Introduction

FBN1-related geleophysic dysplasia (GD2; MIM 614185) is an ultrarare autosomal dominant disorder belonging to the acromelic dysplasia group, which also includes acromicric dysplasia (AD; MIM 102370) and Weill-Marchesani syndrome (WMS; MIM 608328) (1, 2). These conditions are caused by dominant-negative variants specifically clustered in exons 42 and 43 of *FBN1*, which encode the transforming growth factor-β binding protein-like domain 5 (TB5) of fibrillin-1 (3, 4). Fibrillin-1 is a key structural glycoprotein of extracellular matrix microfibrils, providing tissue integrity and regulating the bioavailability of growth factors such as TGF-β (5, 6).

Clinically, GD is characterized by severe short stature, brachydactyly, progressive joint contractures, a characteristic “happy” face, and progressive cardiac valvular thickening that can lead to early death (7–9). Hepatomegaly is a well-described feature, particularly in patients with recessive *ADAMTSL2* mutations (GD1) (7, 10). However, existing literature describes hepatic involvement primarily as mild hepatomegaly or fibrosis in pediatric or young adult cohorts, with no long-term follow-up data on its progression (11–13).

Herein, we report a 76-year-old male patient with FBN1-related GD complicated by late-onset liver cirrhosis, with no other identifiable hepatic risk factors identified. This case presents a novel clinical observation that may point to a potential connection between GD and late-onset cirrhosis in elderly patients, further broadening the recognized phenotypic spectrum of this rare fibrillinopathy.

## 2 Case presentation

A 76-year-old Chinese man was initially referred to the gastroenterology department for evaluation of intermittent abdominal distension that had lasted for 1 year. Liver cirrhosis was incidentally detected on a routine abdominal ultrasound. The attending physician noted the patient’s severe congenital short stature and typical brachydactyly during the initial diagnosis and treatment for liver cirrhosis. These manifestations are not associated with conventional liver diseases, so a consultation with the Department of Endocrinology and Metabolism was requested. The aim of the consultation was further etiological clarification of the systemic malformations. The patient denied accompanying symptoms such as nausea, vomiting, jaundice, or gastrointestinal bleeding. His past medical history was unremarkable, with no history of viral hepatitis, alcohol consumption, hepatotoxic medication use, or metabolic syndrome. He had not received any endocrine-related diagnosis or treatment for his short stature and skeletal malformations before this visit.

**Figure 1:**
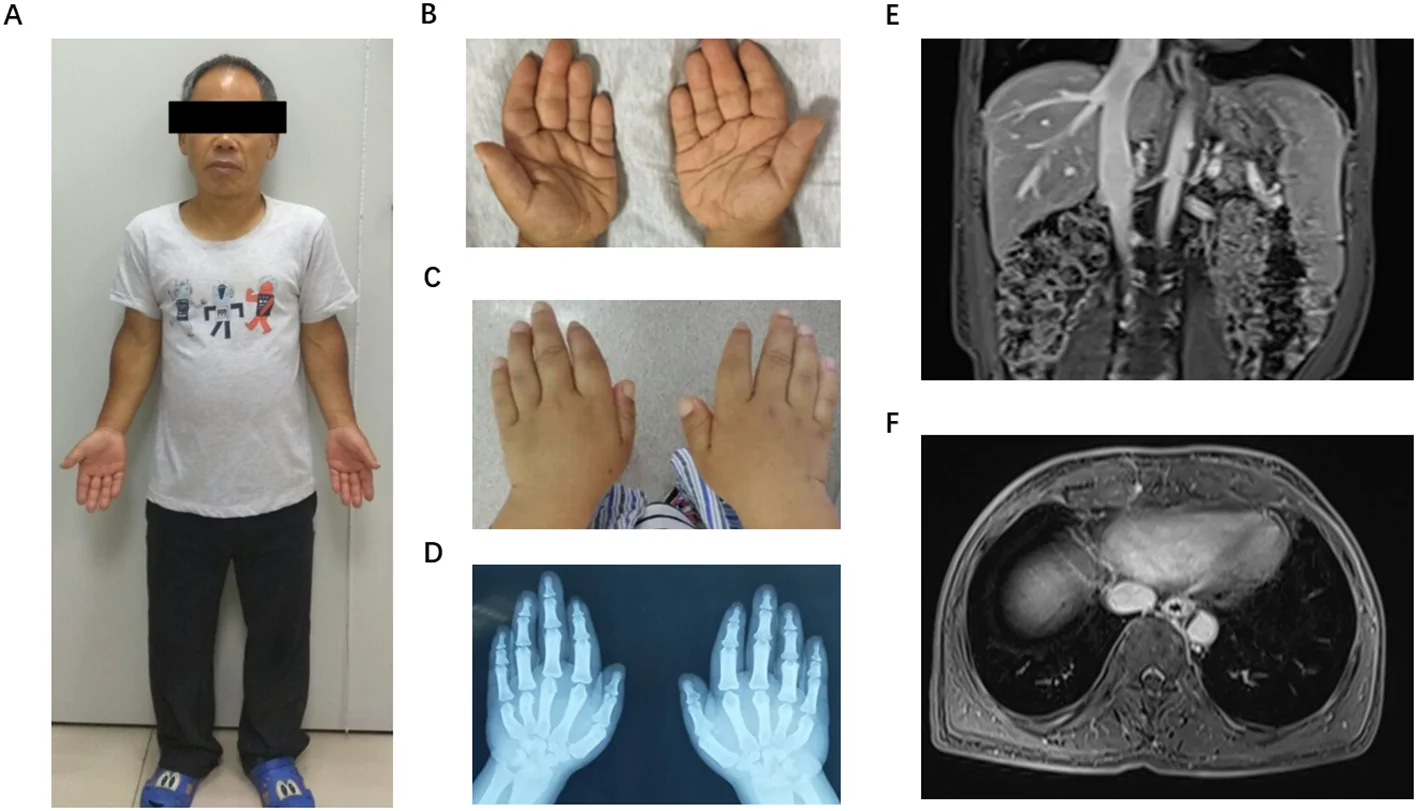
Clinical phenotypes and hepatic magnetic resonance imaging (MRI) findings of the proband. **(A)** General appearance of the proband, presenting with short stature; **(B)** Hand photograph of the proband, showing typical brachydactyly; **(C)** Hand photograph of the proband’s daughter; **(D)** Hand radiograph of the proband’s daughter, demonstrating marked shortening of the phalanges and metacarpals; **(E)** Contrast-enhanced hepatic MRI of the proband, showing manifestations of liver cirrhosis including an irregular hepatic surface and heterogeneous parenchymal signal intensity, with concomitant splenomegaly; **(F)** Contrast-enhanced hepatic MRI of the proband, revealing esophagogastric varices.

Physical examination revealed a conscious, well-appearing man with mild scleral icterus. No stigmata of chronic liver disease (spider angiomata, palmar erythema) were present. Abdominal examination was benign with no hepatosplenomegaly or ascites. Notably, the patient had severe short stature (height 128 cm, **Figure 1A**), with marked shortening of the extremities and bilateral brachydactyly (**Figure 1B**). Joint mobility was normal for his age. His weight was 36 kg, with a BMI of 21.97 kg/m². No signs of endocrine dysfunction such as abnormal thyroid enlargement or abnormal fat distribution were found.

The patient’s 39-year-old daughter had short stature (127 cm) with a similar skeletal phenotype (**Figures 1C, D**). She had a history of congenital cardiac valvular disease and underwent valve replacement at age 36. Liver function tests and abdominal ultrasound performed three years prior to the current presentation were unremarkable; however, no subsequent longitudinal liver surveillance has been completed. Therefore, subclinical or slowly progressive hepatic involvement in the daughter cannot be definitively ruled out based on limited intermittent testing data. The patient’s spouse was healthy, and there was no other family history of skeletal dysplasia, liver disease, or consanguinity.

Laboratory tests showed mild to moderate unconjugated hyperbilirubinemia (total bilirubin 26.4-75.6 μmol/L, direct bilirubin 8.2-13.5 μmol/L) and mild thrombocytopenia (platelets 101-120 × 10^9^/L). Liver enzymes (ALT, AST), synthetic function (albumin, INR), and renal function were normal. Serological workup for viral hepatitis (A, B, C, E) and autoimmune liver disease (ANA, sp100, AMA-M2, LKM, SMA, LC-1) was negative. The serum ceruloplasmin was 207 mg/L (200–600 mg/L), serum iron 44.7 μmol/L (10.6-36.7 μmol/L), ferritin 306 μg/L (30–400 μg/L), transferrin 187.00 mg/dL (200.00-360.00 mg/dL), and transferrin saturation was calculated to be 95.5%. Whole blood lead was 35.90 μg/L (0.00–100.00 μg/L), and both immunofixation electrophoresis and serum protein electrophoresis were normal. Of note, α1-antitrypsin testing was not available at our institution. The endocrinology department completed an endocrine profile assessment. It showed normal thyroid function with TSH, FT3, FT4 within reference ranges, normal pituitary function including GH, IGF-1, ACTH, cortisol and sex hormones, and normal glucose and lipid metabolism indicators. These results ruled out common endocrine causes of short stature such as growth hormone deficiency, hypothyroidism, and congenital adrenal hyperplasia.

Liver elastography revealed a markedly elevated stiffness value of 15.5 kPa. Contrast-enhanced MRI confirmed cirrhosis with an irregular liver surface, heterogeneous parenchyma, splenomegaly, a small amount of ascites, and esophagogastric varices (**Figures 1E, F**).

A genetic etiology was highly suspected based on the comprehensive findings from the joint consultation of gastroenterology and endocrinology departments. The key findings included unexplained liver cirrhosis, typical congenital short stature and brachydactyly, normal endocrine function that ruled out common endocrine etiologies, and familial skeletal malformations. Whole-genome sequencing with Sanger confirmation identified a heterozygous missense variant in exon 43 of the *FBN1* gene (NM_000138.5:c.5284G>A), resulting in a p.Gly1762Ser substitution in the TB5 domain. This variant is classified as pathogenic according to ACMG guidelines, is absent from population databases such as gnomAD, and is a well-established recurrent hotspot mutation for GD/AD (3, 8). The identical mutation was confirmed in his affected daughter. Importantly, the sequencing data also systematically excluded pathogenic variants in genes associated with inherited metabolic liver diseases, including *SERPINA1* (α1-antitrypsin deficiency), *HFE* and non-*HFE* hemochromatosis genes (*HJV, HAMP, TFR2, SLC40A1*), and *ATP7B* (Wilson disease). Although the patient presented with markedly elevated transferrin saturation alongside normal ferritin, this biochemical abnormality can be partially explained by cirrhosis-induced hepcidin dysregulation. Whole-genome sequencing identified no pathogenic variants within all known genes associated with hereditary iron overload. Notably, despite the reassuring overall biochemical and genetic profile, dedicated MRI-based hepatic iron quantification and liver biopsy (the gold-standard modalities for assessing intrahepatic iron deposition) were not performed in this case. Accordingly, secondary iron overload or rare, currently unrecognized genetic or acquired causes of elevated transferrin saturation cannot be completely excluded.

**Figure 2:**
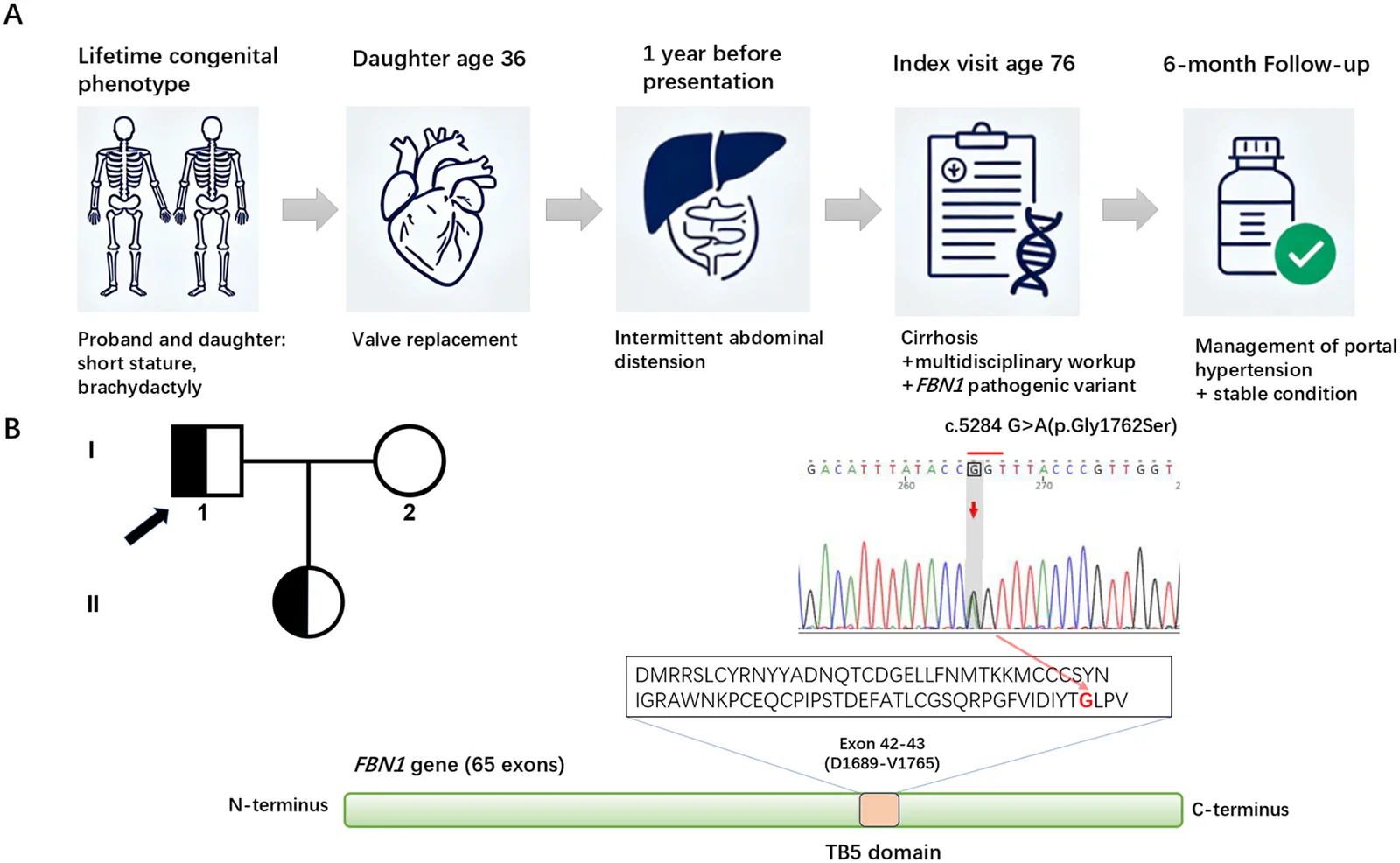
Clinical timeline, pedigree chart, Sanger sequencing electropherogram and schematic diagram of the *FBN1* gene. **(A)** Clinical timeline of the patient. The timeline systematically displays the whole clinical process, including symptom onset, laboratory tests, imaging examinations, genetic testing, treatment and follow-up, which is compiled in accordance with the CARE guidelines for case reports. **(B)** Pedigree chart, Sanger sequencing electropherogram and schematic diagram of the *FBN1* gene. Half-filled symbols indicate heterozygous mutation carriers, and the black arrow denotes the proband. In the Sanger sequencing electropherogram, the red arrow points to the pathogenic mutation site (c.5284G>A). The schematic diagram of the *FBN1* gene highlights the TB5 domain (TGFβ-binding protein-like domain 5), the mutation region identified in this case.

Of note, the patient presented with persistent mild-to-moderate unconjugated hyperbilirubinemia throughout the clinical course. The etiology of this biochemical abnormality remains unclear, and its direct correlation with FBN1-related GD has not been validated in existing literature. Whether this hyperbilirubinemia represents a secondary hepatic manifestation of GD, a coincidental benign biochemical variant, or an unrecognized associated phenotypic feature requires further long-term observation and additional clinical evidence. The full clinical course of the patient is summarized in **Figure 2A**. The pedigree chart, Sanger sequencing results and schematic diagram of the *FBN1* gene are shown in **Figure 2B**.

Standardized hepatological care was administered, focusing on the management of portal hypertension. Upper gastrointestinal endoscopy was recommended to assess esophagogastric varices and guide primary prophylaxis. Mild ascites was managed via dietary sodium restriction and serial volume monitoring. Surveillance for hepatocellular carcinoma was conducted semiannually using liver ultrasound and serum alpha-fetoprotein.

The patient stated that he and his daughter had lifelong short stature; his daughter had congenital valvular heart disease and underwent surgical repair. Notably, the diagnosis of FBN1-related geleophysic dysplasia was identified by genetic testing after the incidental finding of liver cirrhosis.

## 3 Discussion

To our knowledge, this case represents the first report of cirrhosis with portal hypertension in an elderly patient with molecularly confirmed FBN1-related GD. The 76-year-old male patient carried a heterozygous p.Gly1762Ser mutation in the TB5 domain of the *FBN1* gene, accompanied by severe short stature, acromelic shortening and late-onset liver cirrhosis, without conventional risk factors for liver disease.

Hepatic involvement in GD, particularly in the FBN1-related form (GD2), has generally been considered mild and non-progressive. While hepatomegaly is seen in about one-third of patients, its long-term evolution has not been well documented (7, 11). In the large natural history study by Marzin et al., hepatomegaly was more frequent in patients with *ADAMTSL2* mutations, and progressive liver disease was not a prominent feature in their cohort (7). The present case raises the hypothesis that the underlying ECM defect might create a theoretical susceptibility to hepatic fibrotic changes, analogous to the fibrotic lesions observed in cardiac valves and airways of individuals with GD (8, 9, 14).

The pathogenesis of liver fibrosis in GD is likely closely related to dysregulation of the TGF-β signaling pathway, a core element of GD pathophysiology (3, 4, 6). As a theoretical hypothesis for this case, liver fibrosis in the present patient is speculated to be associated with this abnormal pathway. Mutant fibrillin-1, particularly proteins carrying TB5 domain mutations such as p.Gly1762Ser, disrupts the microfibrillar network structure and fails to properly bind and sequester latent TGF-β complexes (3, 6). This leads to elevated levels of bioactive TGF-β, the most important profibrotic cytokine in the liver, which activates hepatic stellate cells and drives excessive ECM deposition (15). This mechanism provides a theoretical explanation for the silent, non-inflammatory liver changes seen in this patient, who maintained normal transaminases and preserved hepatic synthetic function at the time of cirrhosis detection.

This case also reveals remarkable clinical heterogeneity associated with the identical *FBN1* mutation within the same family. The proband and his daughter carry the exact same p.Gly1762Ser mutation and share similar phenotypes of short stature and acromelic shortening. However, the daughter’s clinical presentation is dominated by cardiac valvular disease, requiring valve replacement surgery in her 30s, and no evidence of hepatic involvement was noted at that time. This intrafamilial variability fully reflects the influence of genetic modifiers, environmental factors, or differential long-term cumulative effects of ECM remodeling in different tissues (16). Given the intrafamilial phenotypic variability of this variant, routine multi-organ surveillance including liver assessment may be considered for asymptomatic carriers; however, there is no evidence to confirm subclinical hepatic fibrosis in the proband’s daughter at present.

**Figure 3:**
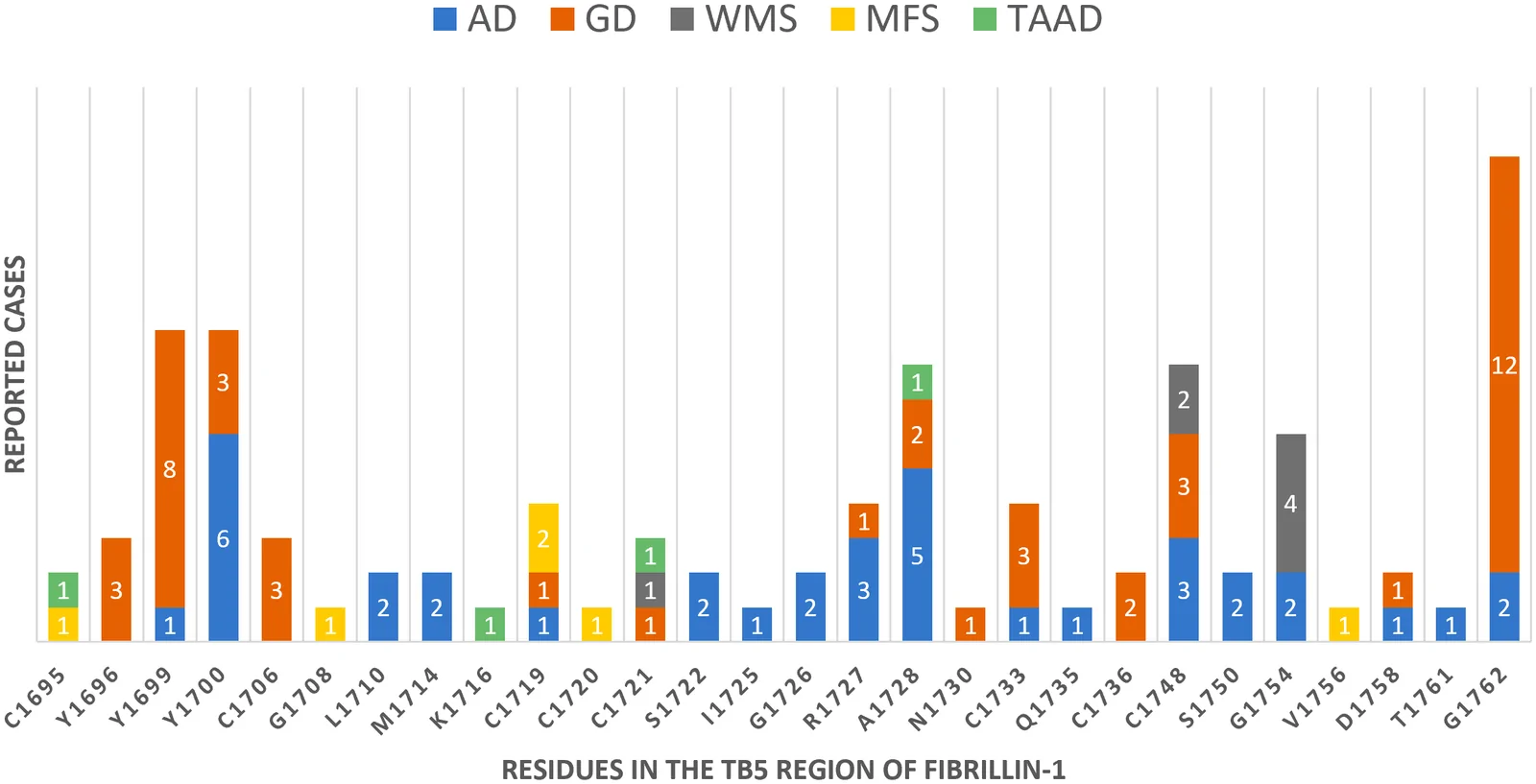
Summary of disease-associated amino acid variants in the TB5 domain of the *FBN1* gene. All variants associated with acromelic dysplasia spectrum disorders are in-frame missense variants within the TB5 domain, and the p.Gly1762Ser (c.5284G>A) variant identified in this case is one of the most common recurrent hot-spot mutations linked to GD and AD. Different colors represent distinct FBN1-related disorders associated with each mutated amino acid residue in this domain. AD, acromicric dysplasia; GD, Geleophysic dysplasia; WMS, Weill-Marchesani syndrome; MFS, Marfan syndrome; TAAD, thoracic aortic aneurysm and dissection.

As shown in **Figure 3**, we summarized the genotype-phenotype correlations within the TB5 domain of the *FBN1* gene. All acromelic dysplasia-associated *FBN1* mutations are in-frame amino acid variants within this domain. Notably, multiple amino acid residues within the TB5 domain exhibit significant allelic heterogeneity, with the same mutation manifesting as GD, AD, or even broader clinical spectra in different patients (2, 3). This incomplete genotype-phenotype correspondence further confirms the high degree of phenotypic heterogeneity of TB5 domain mutations and suggests that, beyond the mutation itself, other genetic or environmental modifiers play important roles in shaping the final clinical presentation.

In clinical practice, for patients with unexplained late-onset liver cirrhosis presenting with syndromic features such as short stature and acromelic shortening, rare genetic disorders such as GD should be included in the differential diagnosis. Accurate differentiation from other FBN1-related disorders is crucial for prognosis assessment and family management.

Marfan syndrome (MFS) is caused by mutations in other regions of *FBN1*, as well as by truncating mutations in the TB5 domain, and presents with a skeletal phenotype opposite to that of GD, namely tall stature and arachnodactyly, with aortic root disease as its major cardiovascular manifestation rather than liver cirrhosis (5). Weill-Marchesani syndrome (WMS) also features short stature, but is set apart by ocular abnormalities such as microspherophakia and ectopia lentis, neither of which were present in our patient (1).

The clinical overlap between AD and GD warrants careful consideration. As shown in **Figure 3**, identical *FBN1* variants produce divergent phenotypes across patients, implying AD may constitute a milder or earlier manifestation within a unified GD–AD disease spectrum. Historically, AD was thought to spare progressive cardiac and hepatic lesions (2, 7). However, shared genetic drivers and molecular therapeutic targets, combined with the late-onset cirrhosis identified in our proband, raise concerns regarding long-term hepatic complications in AD patients. Though these disorders are exceedingly rare, liver-related complications may emerge in older carriers of TB5 domain variants. Given limited clinical evidence, hepatic surveillance may be cautiously recommended only for TB5 domain variant carriers rather than implemented universally.

By the same logic, routine long-term liver monitoring is not mandated for all GD patients. Serial liver biochemistry, liver elastography and abdominal ultrasound can facilitate early identification of abnormal liver findings in middle-aged and elderly GD patients at higher risk, allowing timely clinical intervention if hepatic abnormalities are detected. In addition, first-degree relatives of confirmed GD probands should receive clinical evaluation and *FBN1* genetic testing to identify asymptomatic variant carriers, who require baseline multi-organ assessment covering the heart, liver and skeleton. Given the variable expressivity of TB5 domain variants, this strategy avoids delayed diagnosis and irreversible organ complications in at-risk family members.

As a single case report, this study has certain limitations. First, the diagnosis of liver cirrhosis was established based on liver elastography and contrast-enhanced MRI rather than liver biopsy. The absence of histopathological evidence weakens the inference that liver cirrhosis is linked to FBN1-related GD. However, the comprehensive exclusion of other etiologies, strong genetic evidence, and typical family history make the diagnosis highly reliable. Second, the follow-up period is relatively short (6 months), precluding observation of the long-term effects of liver-directed supportive management on GD-related liver cirrhosis. Future multi-center studies with larger sample sizes and long-term follow-up data are needed to clarify the true incidence, risk factors, and effective interventions for liver cirrhosis in GD patients.

## 4 Conclusions

We report a case of late-onset liver cirrhosis in an elderly patient with FBN1-related GD. This clinical finding raises the possibility of a potential clinical association between GD-related hepatic manifestations and late-onset cirrhosis with portal hypertension in aged individuals, while longitudinal hepatic data are lacking to verify progressive liver damage over time. This observation broadens the known phenotypic spectrum of GD and suggests individualized long-term multi-organ follow-up may be considered for at-risk patients rather than universal surveillance for all affected individuals. Clinicians should consider FBN1-related genetic disorders and relevant genetic testing when managing patients with unexplained cirrhosis combined with short stature and familial skeletal malformations.

## Data Availability

The datasets presented in this study can be found in online repositories. The names of the repository/repositories and accession number(s) can be found below: https://figshare.com/, 10.6084/m9.figshare.31476814.
